# Cost of using a patient tracer to reduce loss to follow-up and ascertain patient status in a large antiretroviral therapy program in Johannesburg, South Africa

**DOI:** 10.1111/j.1365-3156.2010.02512.x

**Published:** 2010-06

**Authors:** Sydney Rosen, Mpefe Ketlhapile

**Affiliations:** 1 Center for Global Health and Development, Boston UniversityBoston MA, USA; 2 Health Economics and Epidemiology Research Office, Wits Health ConsortiumJohannesburg, South Africa; 3 Faculty of Health Sciences, University of the WitwatersrandJohannesburg, South Africa

**Keywords:** highly active antiretroviral therapy, loss to follow-up, cost, South Africa

## Abstract

**Objective:**

To evaluate a pilot intervention to engage a patient tracer to follow up lost patients at a large public clinic in South Africa.

**Methods:**

A social worker spent 4 months contacting by telephone a random sample of patients who had initiated antiretroviral therapy (ART) at least 6 months earlier and were ≥1 month late for a scheduled visit. The tracer was authorized to assist patients to return to care if needed. Cost was calculated from the perspective of the clinic.

**Results:**

The tracer was able to determine the final status of 260 of a sample of 493 lost patients. Of the 260, 55 (21%) had died, 56 (21%) were still on ART at the same site, 79 (30%) reported transferring to another site and 70 (27%) had discontinued treatment. Among those discontinuing, commonly cited reasons were relocation (*n* = 18, 26%), traditional medicine or religious beliefs (*n* = 11, 16%), fear of disclosure or other family barriers (*n* = 9, 13%), and employment obstacles (*n* = 7, 10%). Twenty patients returned to care at the original site as a result of the intervention, at an average cost of $432 per patient returned.

**Conclusions:**

A patient tracer was an effective way to determine the final status of lost patients and succeeded in returning some to care, but the cost per patient returned was high. Better information systems allowing sites to track deaths and transfers would greatly improve the efficiency of loss to follow-up interventions.

## Introduction

As large, public sector programs for providing antiretroviral therapy (ART) for HIV/AIDS have expanded and matured in sub-Saharan Africa, high rates of patient attrition from the programs have become apparent. While much attrition is due to mortality, loss to follow-up – the disappearance of the patient from the program, for no reported reason – is even more common. A systematic review of cohort studies from the region published between 2007 and 2009 found that loss to follow-up comprised an average of 59% of all reported attrition in the first year of treatment (Fox & Rosen 2010). Roughly 25% of all patients who initiated ART had been lost to follow-up or had died 2 years after starting treatment.

Loss to follow-up is a difficult issue to study and to address, because the patients involved have opted out of care, either voluntarily or involuntarily, and thus cannot readily be reached. Providers typically do not know whether a lost patient has died; transferred to a new treatment site; been unable to remain in care due to economic, social or psychological barriers; or simply chosen to discontinue treatment. In a study of reasons for loss to follow-up among patients on ART for 18–48 months at a public hospital in Johannesburg, we found that some patients who had dropped out of the treatment program were willing to return to care but needed help in overcoming a relatively minor obstacle, such as the inability to provide the documentation that they believed would be required by the clinic or a fear of being chastised by the clinic staff. Others faced more structural but also solvable problems, such as lack of a small amount of money for transport to the clinic (Miller *et al.* 2010).

One intervention that has been tried by a number of treatment sites is to try to contact lost patients by cell phone and/or home visits, for the purpose of either determining their status or, more ambitiously, of helping them to return to care. These experiments with ‘patient tracers’ have often proven effective in determining the status of lost patients (Geng *et al.* 2008). Success in returning patients to care has been more mixed (Billy *et al.* 2007; Ochieng *et al.* 2007; Krebs *et al.* 2008; Tweya *et al.* 2009). In Kenya, for example, phone calls and home visits resulted in 65% and 49% of traced patients returning to care in an urban and rural area, respectively (Ochieng *et al.* 2007). A follow-up intervention in Malawi, however, resulted in only 19% of all lost patients returning to care (Tweya *et al.* 2009). In Zambia, an average of 18 home visits to lost patients in an urban setting was required per patient who ultimately returned to care (Krebs *et al.* 2008).

As the study of home visits in Zambia suggests, many contacts with patients who cannot be returned to care may be required for each one who does return. For large, public-sector treatment facilities in resource-constrained settings, this raises the issues of feasibility and affordability, both in an absolute sense and relative to other potential interventions for reducing loss to follow-up (Losina *et al.* 2009). To help one treatment program in South Africa address this issue, we conducted a cost-effectiveness analysis of a pilot ‘patient tracer’ intervention that the site implemented over a 4-month period in 2009.

## Methods

### Study site

The intervention was implemented at Themba Lethu Clinic, the HIV/AIDS clinic of Helen Joseph Hospital in Johannesburg, South Africa. Themba Lethu, one of the oldest and largest providers of antiretroviral treatment in South Africa, provides some 12 000 adult patients with ART. It is funded primarily by the Gauteng Province Department of Health, with technical and financial support from Right to Care, a non-governmental partner of the U.S. President's Emergency Plan for AIDS Relief (PEPFAR). The site has been described in numerous publications (Sanne *et al.* 2005; Maskew *et al.* 2007; Rosen *et al.* 2007, 2008a,b,c; Dalal *et al.* 2008).

Antiretroviral therapy patient loss to follow-up at Themba Lethu Clinic is in line with other estimates from the region. Of roughly 7500 patients initiated on ART in the first 3 years of the program (April 1, 2004–March 31, 2007), 16.4% had been lost to follow-up by March 31, 2008. Nearly 40% of these were lost in their first 3 months on ART, and the rest over the following 4 months to 4 years (Majuba *et al.* 2009). An analysis of a smaller cohort of 200 patients initiated on ART at Themba Lethu in 2005 estimated a 24-month loss to follow-up rate of 29% (Long *et al.* 2008). Clinic staff believes that patient retention has improved since then, as has the tracking of patient mortality, but loss to follow-up almost certainly remains in double digits even for the most recent patient cohorts.

### Description of intervention

The intervention was limited to adult patients who had initiated ART at Themba Lethu Clinic in or after April 2004, had been on ART for at least 3 months at their last clinic visit, were between 1 and 12 months late for their next scheduled visit or medication pickup, and were not known to have died or transferred to another treatment site. All patients meeting these criteria were identified through the clinic's electronic information system and then stratified into four groups on the basis of duration on ART (less than or more than 1 year as of last clinic visit) and CD4 count at most recent test (less than or more than 350 cells/mm^3^). Finally, patients in each group were selected at random for inclusion in the intervention.

Although Themba Lethu Clinic has for several years employed a counsellor to track patients lost to follow-up, this counsellor is generally limited to making one or two phone calls to the contact numbers provided by patients to determine their status and is not authorized to provide any specific assistance to help patients return to care. The intervention evaluated by this study engaged a researcher with training in social work to serve as an enhanced patient tracer on an 80% time basis (4 days/week) for 4 months. The researcher engaged for this purpose was a university graduate who was employed by a research unit attached to the study clinic. She was very familiar with the clinic and had implemented other studies at the site, including conducting qualitative interviews with patients who had been lost to follow-up. During the implementation of the intervention, the researcher was seconded to the study clinic and operated as a member of the clinic staff.

In the role of patient tracer, the researcher was instructed to use all available contact information to locate patients; discuss the patient's situation by phone or in person; and, following pre-established guidelines, assist the patient to return to care. Assistance allowed by the intervention's guidelines included making an appointment at the clinic on the patient's behalf; meeting the patient for the appointment and assisting in the consultation if requested; helping to obtain documentation required to continue treatment; finding and referring the patient to another treatment facility closer to the patient's home or open at different times; helping the patient to disclose his/her HIV or treatment status to family members; providing other information or referrals as requested; and/or providing up to R100 (approximately $11) per patient for transport fares to and from the clinic. The intention of providing transport funds was to allow the patient to make one additional roundtrip visit to the clinic, in the hope that the clinic staff could assist the patient in devising a longer-term solution to the lack of transport funds, such as transferring to a closer facility or applying for a social support grant.

The intervention was implemented between February 10 and June 10, 2009. Both initial and follow-up contacts were made in the first 3 months of the intervention. The fourth month was limited to follow-up contacts only, ensuring that all patients who were contacted had at least 1 month to return to care before the intervention was completed and evaluated.

### Data analysis

The patient tracer maintained detailed records of all attempts to contact patients, successful contacts, patient status, recommendations or assistance given, and costs incurred. Aggregate outcomes for evaluation included number of previously lost-to-follow-up patients known to have returned to care at the study site or another facility as a result of the intervention and number of previously lost-to-follow-up patients whose status was newly determined by the intervention. The average cost to achieve each outcome was then estimated. Intervention costs included the tracer's salary and benefits, the cost of a workstation at the site, and equipment, phone and transport charges. To consider the costs if the tracer's salary had been that of nurse at the clinic, rather than a researcher employed externally, a second analysis was run using the current salary of a government junior enrolled nurse.

## Results

### Sample

**Figure 1:**
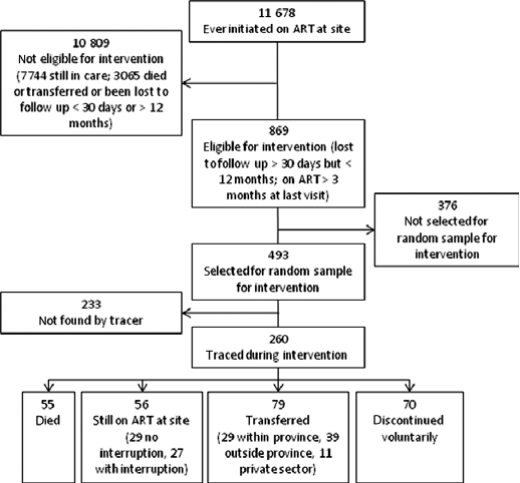
Status of patients in intervention.

Of approximately 11 700 patients who had started ART at the study clinic by the end of September 2008 and could thus have been on treatment for at least 3 months at the time of sample selection, 869 patients met all the inclusion criteria for the intervention as of January 8, 2009, as illustrated in Figure 1. A random sample of 493 (57%) of these patients was selected for follow-up by the tracer. Over 4 months, the tracer was able to reach or learn the status of 260 (53%) patients. The remaining 233 (47%) could not be located using contact information provided to the clinic by the patients. As shown in Table 1, patients whose last reported CD4 count exceeded 350 cells/mm^3^ were more likely to be located by the tracer than those with lower CD4 counts at their last visit.

**Table 1:** Proportion of sampled patients located by tracer, by duration on ART and last CD4 count

Duration on ART at time of loss to follow-up	Number traced/number in sample (%)		
	
Last reported CD4 count (cells/mm^3^)	<350	>350	All
On ART <1 year	59/107 (55%)	9/10 (90%)	68/117 (58%)
On ART >1 year	120/260 (46%)	72/116 (62%)	192/376 (51%)
All	179/367 (49%)	79/126 (63%)	260/493 (53%)

ART, antiretroviral therapy.

Among those reached, all but one patient were contacted by telephone only; at the patient's request, one contact was made in person, at the patient's home. The tracer made a total of 883 phone calls in the 4-month period, or an average of 1.8 calls per patient in the sample. Each patient was called between 0 and 8 times. One hundred and forty patients did not provide any contact information and could not be called at all (0 calls). Among the 353 who were called at least once, the average number of calls/patient was 2.5. Of these, 93 patients were not found by phone (calls not answered or answered by someone who did not know the patient or did not know the patient's location or status). The status was determined for the remaining 260.

### Tracing results

The status of the 260 patients who could be traced is shown in Table 2. Approximately one fifth of the sample was reported by household members to have died. Another fifth told the tracer that they were still on treatment at the same facility; of these, roughly half said that they had not missed any appointments, indicating an error in the site's record-keeping. The other half had interrupted their treatment temporarily, justifying the notation of lost to follow-up in their records, but returned to the clinic during the intervention period.

**Table 2:** Status of patients traced during intervention

Status	*n*	%
Died (as reported by person answering contact phone number)	55	21
Still on ART at site	56	21
No interruption (records incorrect)	29	52
With interruption; temporary loss to follow-up	27	48
Transferred to a different site	79	30
Local treatment site (within Gauteng Province)	29	37
Distant treatment site (different province or country)	39	50
Private sector provider	11	13
Discontinued treatment	70	27
Total	260	100

ART, antiretroviral therapy.

Nearly a third of the sample (30%) reported that they had transferred to another ART facility, either nearby or in a different province or country. Those transferring locally generally cited transport cost as the reason for transferring, though several said that they were now attending a nearby clinic that was open on Saturdays to avoid conflict with work schedules. Most of those who transferred to more distant sites indicated that they had returned to their original homes after living in Johannesburg temporarily.

Finally, the remaining 27% of patients traced had discontinued treatment for a variety of reasons, as shown in Table 3. Approximately a quarter of these patients reported that they had left Johannesburg and had not re-started treatment at a new facility, though some indicated that they still intended to do so. The next largest group – 16% of the total – cited traditional medicine or religious beliefs as their reason for discontinuing treatment. Among these, 4 of the 11 patients said that they believed they had been cured of HIV and no longer needed treatment. A number of patients were unwilling to remain in care due to fear of disclosure of their HIV status to their partners or families or for other family-related reasons. Several others expressed fear of losing their jobs if they took time off to attend the clinic. A variety of other reasons were cited by smaller numbers of patients, as shown in Table 3.

**Table 3:** Patients' stated reasons for discontinuing treatment

Reason	*n*	%
Relocated, did not re-start treatment in new location	18	26
Traditional medicine, religious beliefs, religious training	11	16
Fear of disclosure or other family barriers	9	13
Conflict with employment obligations	7	10
Undergoing TB treatment	6	9
Cost of transport	6	9
Side effects	3	4
Too ill to visit clinic	1	1
Believed ART would prevent pregnancy	1	1
No reason provided	8	11

ART, antiretroviral therapy.

Over the course of the 4-month intervention, the tracer was able to persuade 20 of the 97 patients (21%) who had voluntarily discontinued treatment prior to being contacted by the tracer to return to the clinic. Each of these 20 patients completed at least one clinic visit during the intervention period. An additional 27 patients (28%) said that they would like to return to care and allowed a visit to be scheduled for them but did not complete a visit before the end of the intervention. Several others indicated that the tracer's call would motivate them to resume treatment at a new site, but we were not able to confirm that they did so.

### Intervention cost and cost per patient returned to care

The main cost of the intervention was the salary of the tracer, who was employed for 4 months. Costs in 2009 USD are shown in Table 4, using the average exchange rate over the intervention period (February–May 2009, R9.36/$1.00).

**Table 4:** Intervention costs over 4 months

Item	Total cost	Cost/month
Tracer's salary and benefits	$7306	$1827
Office expenses and service	$1127	$282
Communications and supplies	$176	$44
Patient transport reimbursement	$23	$6
Total	$8632	$2158
Alternative tracer's salary and benefits (junior nurse)	$4224	$1056

For the 20 patients returned to care by the tracer, the cost per patient for the intervention was $432/patient. This estimate does not take into account other outcomes of the intervention, such as the improvement in the accuracy of the clinic's records through the ascertainment of the true status of patients recorded as lost to follow-up; the possible return to care by some patients as a result of the intervention after the intervention period ended; and the possible return to care at other sites of patients for whom the tracer's contact provided the necessary motivation. The average cost per patient attempted to be traced in the intervention, including those who could not be found through tracing, was $18.

Because the salary of the patient tracer in this evaluation was equivalent to that of a professional (senior level) nurse at the clinic, we also considered the cost of the intervention had the tracer been a junior enrolled nurse instead. In this case, assuming the same results of the intervention, the cost per patient returned to care would have fallen to $262, and the average cost per patient attempted to be traced would have been $11. We cannot know, however, whether a junior nurse, who would likely have been less experienced than our tracer and lacked training in social work, would have achieved the same results.

If patients who had died, transferred to another site, or been mistakenly reported as lost to follow-up due to record-keeping errors could have been removed from the tracer's original sample prior to starting the intervention, the number of patients on the tracer's roster would have fallen by 39%, and the cost per patient returned to care to $265 using the tracer's actual salary or $161 using a junior nurse's salary.

## Discussion

Over a 4-month period, a dedicated patient tracer working 4 days/week was able to track 493 patients and determine the final status of 260 patients previously recorded as lost to follow-up in clinic records. Just over half (51%) of these patients were still on treatment, at least by self-report, at either the same site or at a different site. Another 21% had died. Only 27% of patients admitted to having stopped treatment entirely, with reasons ranging from relocation to traditional medicine to fear of disclosure.

Although the intervention cost relatively little to implement, the modest number of patients known to have been returned to care by it meant that it was relatively expensive per patient returned to care. The average cost per patient returned to care, $432, is equivalent to the cost of nearly a year of treatment (11.3 months) with first-line therapy at the same site (author's data). A more junior tracer would have cost less, but the cost per patient returned to care ($262) would still have been high. In an era of tight budgets for HIV/AIDS treatment, particularly in South Africa (Ntutu 2009), spending the equivalent of even half a year's worth of treatment to track down an errant patient may seem unwise, if not unfair to patients on the waiting list for ART who have not yet had the opportunity to initiate treatment at all. Improving the effectiveness of the intervention – increasing the yield of lost patients who are returned to care – would thus seem essential for this type of intervention to be advocated on a larger scale. On the other hand, when the total cost of the intervention is spread across all the patients in the intervention sample who could be found, the cost per patient fell to $18, and thus represented only a small increase in the average cost of treatment per patient. If the intervention were implemented year-round at the same monthly cost, the average additional cost per patient in care at the study site would be just $3/year, <1% increase in the average cost of ART at this site (author's data).

What could be done to improve the intervention? The most striking aspect of our results is the large proportion of patients who had either died or reported themselves to have transferred, and were thus only lost to follow-up from the perspective of the originating clinic. Greater capacity to trace patients from one site to another is urgently needed. The combination of the tremendous mobility of South African society, in which large numbers of people routinely relocate between provinces in response to work opportunities; the high cost of seeking treatment in terms of both transport fares and time; and the pressures of traditional culture and fear of disclosure make it inevitable that patients will transfer from one treatment site to another frequently and often without warning. An information system that allows the originating site to determine that a patient has transferred to another clinic would immediately cut loss to follow-up rates dramatically. Better reporting of deaths would have a similar effect; in South Africa this can be achieved by online searching of the national death registry (Fox *et al.* 2010). Both changes would allow a patient tracer to concentrate on patients who are truly lost to follow-up and can potentially benefit from an intervention, rather than wasting time trying to locate those who have died or transferred. Excluding from the original sample the deaths and transfers that were reported to the tracer would have reduced the cost per patient returned to care to $265, much less than was actually spent. An effective system for recording deaths and transfers when they happen would likely have screened out even more of the original sample, making the tracer's efforts that much more effective and further reducing the cost per patient returned.

A significant minority of patients who discontinued treatment or transferred to another local clinic indicated that the main obstacle they faced was conflict with employment. The study site is open only on weekdays and during working hours. Many patients who use the public sector have wage jobs that do not allow time off for clinic visits. Others resist taking time off for fear that it will reveal their HIV status to their employers. A simple solution for these patients is to open the clinic in the evenings and/or on Saturdays. While clinic operating costs may be slightly higher during these times due to overtime salary requirements, the additional hours may also reduce waiting times for all patients and extend overall treatment capacity. Patients requiring weekend visits may also be willing and able to pay a small fee for this service, an idea that is under consideration at the study site.

In conclusion, this intervention demonstrated that a patient tracer can successfully track down and determine the status of approximately half the patients recorded as lost to follow-up at a large public sector clinic and can return a handful of them to care. The cost per patient returned to care will be high, however, unless better information systems are implemented to allow patients who have transferred or died to be excluded from the intervention. The cost per patient treated by the clinic is modest, and if most transfers and deaths can be screened out and the tracer supported over a longer period of time, this intervention may provide one useful strategy in the effort to improve long-term patient retention in ART programs.
